# Mitochondrial Mislocalization Underlies Aβ42-Induced Neuronal Dysfunction in a *Drosophila* Model of Alzheimer's Disease

**DOI:** 10.1371/journal.pone.0008310

**Published:** 2009-12-15

**Authors:** Kanae Iijima-Ando, Stephen A. Hearn, Christopher Shenton, Anthony Gatt, LiJuan Zhao, Koichi Iijima

**Affiliations:** 1 Laboratory of Neurogenetics and Pathobiology, Thomas Jefferson University, Philadelphia, Pennsylvania, United States of America; 2 Department of Biochemistry and Molecular Biology, Farber Institute for Neurosciences, Thomas Jefferson University, Philadelphia, Pennsylvania, United States of America; 3 Microscopy Facility, Cold Spring Harbor Laboratory, Cold Spring Harbor, New York, United States of America; Brigham and Women's Hospital/Harvard Medical School, United States of America

## Abstract

The amyloid-β 42 (Aβ42) is thought to play a central role in the pathogenesis of Alzheimer's disease (AD). However, the molecular mechanisms by which Aβ42 induces neuronal dysfunction and degeneration remain elusive. Mitochondrial dysfunctions are implicated in AD brains. Whether mitochondrial dysfunctions are merely a consequence of AD pathology, or are early seminal events in AD pathogenesis remains to be determined. Here, we show that Aβ42 induces mitochondrial mislocalization, which contributes to Aβ42-induced neuronal dysfunction in a transgenic *Drosophila* model. In the Aβ42 fly brain, mitochondria were reduced in axons and dendrites, and accumulated in the somata without severe mitochondrial damage or neurodegeneration. In contrast, organization of microtubule or global axonal transport was not significantly altered at this stage. Aβ42-induced behavioral defects were exacerbated by genetic reductions in mitochondrial transport, and were modulated by cAMP levels and PKA activity. Levels of putative PKA substrate phosphoproteins were reduced in the Aβ42 fly brains. Importantly, perturbations in mitochondrial transport in neurons were sufficient to disrupt PKA signaling and induce late-onset behavioral deficits, suggesting a mechanism whereby mitochondrial mislocalization contributes to Aβ42-induced neuronal dysfunction. These results demonstrate that mislocalization of mitochondria underlies the pathogenic effects of Aβ42 *in vivo*.

## Introduction

Alzheimer's disease (AD) is a progressive neurodegenerative disease without effective therapies. Pathologically, AD is defined by an extensive loss of neurons and by formation of two characteristic protein deposits, extracellular amyloid plaques (APs) and intracellular neurofibrillary tangles (NFTs). The major components of APs and NFTs are the 40 or 42 amino acid amyloid-β peptides (Aβ40 or Aβ42) and the hyperphosphorylated microtubule associated protein tau, respectively [1].

Molecular genetic studies of early-onset familial AD patients have identified causative mutations in genes encoding APP and presenilins (PS1 and PS2), and these mutations increase Aβ42 production and/or Aβ aggregation [2]. Aβ42 is highly toxic to cultured neurons and causes memory deficits and neurodegeneration in animal models overproducing human Aβ42 [3]. Thus, Aβ42 is thought to play a causative role in the pathogenesis of AD [4].

Several lines of evidence indicate that mitochondrial function is impaired in the brains of AD patients [5], [6], [7], [8]. Markedly reduced levels of mitochondrial proteins and activities and increased abnormal and damaged mitochondria have been reported in AD brains [8], [9], [10], [11]. Whether mitochondrial dysfunctions are merely a consequence of AD pathology or are early seminal events in AD pathogenesis remains to be determined.

In order to identify genes and pathways that are involved in Aβ42-induced toxicity *in vivo*, we are utilizing *Drosophila* as a model system. To produce human Aβ42 in the secretory pathway of fly brain neurons, the Aβ42 peptide sequence is directly fused to a secretion signal peptide at the N-terminus. Using a GAL4-UAS transgene expression system [12], Aβ42 peptide was expressed in the fly brain. Mass spectrometry analysis revealed that this construct produces the intact Aβ42 peptide in the fly brain [13], [14], and immuno-electron microscopy analysis showed that expressed Aβ42 was distributed in the secretory pathways in neurons in the fly brains [14]. These Aβ42 flies show late-onset, progressive short-term memory defects, locomotor dysfunctions, neurodegeneration, and premature death, accompanied by formation of Aβ42 deposits [13], [14]. This or similar *Drosophila* models have been used to study mechanisms underlying neurotoxicity of Aβ42 *in vivo* [3], [15], [16], [17], [18], [19], [20], [21], [22], [23].

Using this *Drosophila* model [13], [14], here we have demonstrated that mitochondrial mislocalization underlies the pathogenic effects of Aβ42 *in vivo*.

## Results

### Mitochondria Are Reduced in the Axons and Dendrites in Aβ42 Fly Brain

**Figure 1:**
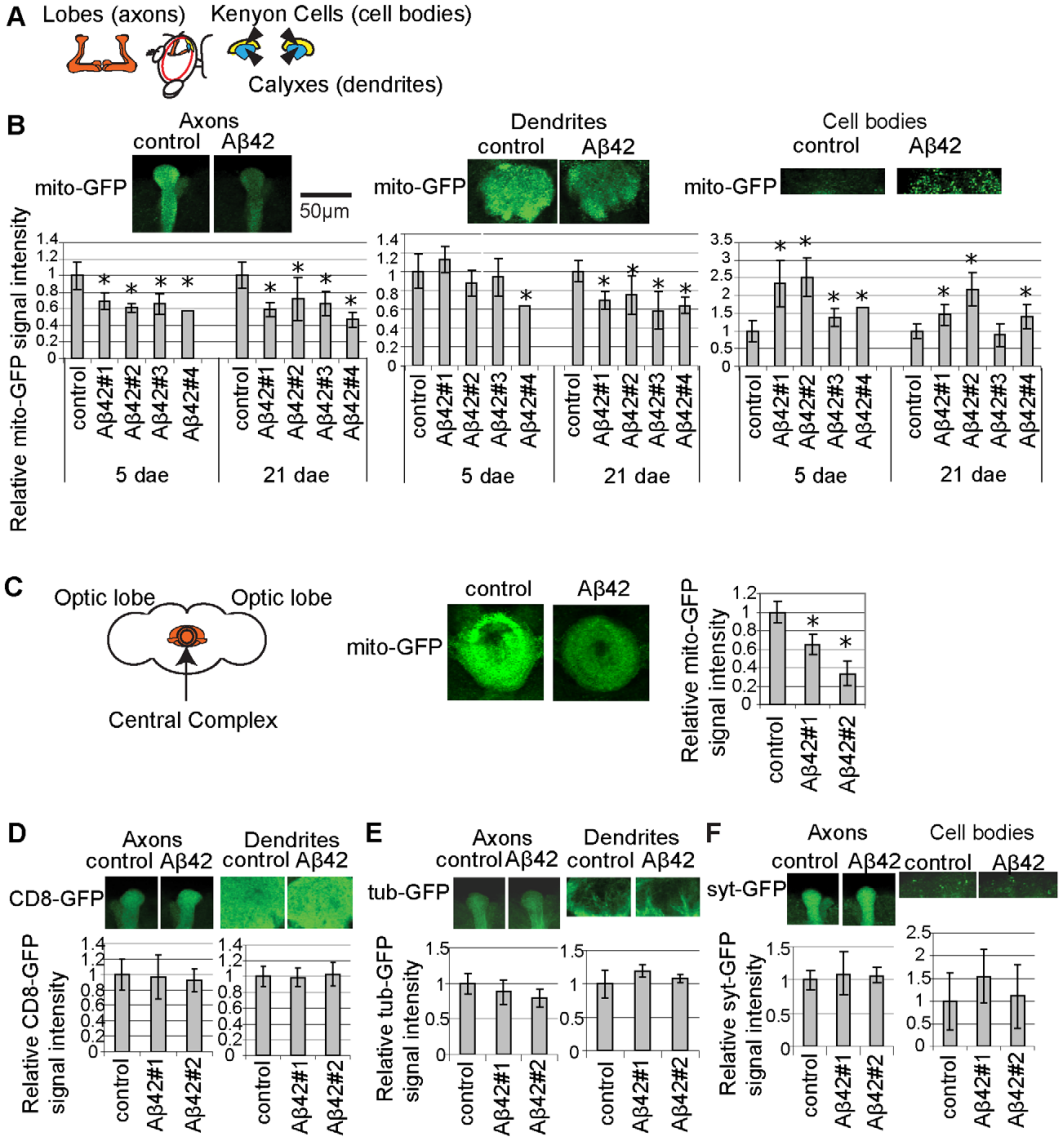
Mitochondria are reduced in the axons and dendrites in Aβ42 fly brain. (A) A schematic view of the mushroom body. (B) Signal intensities of mito-GFP in axon bundle tips, dendrites, and cell bodies of the mushroom body structure in control and Aβ42 flies at 5 days after eclosion (dae) and 21 dae. Ratios relative to control are shown (mean±SD, n = 6–10; *, p<0.05, Student's t-test). Representative images at 21 dae are shown at the top. (C) Signal intensities of mito-GFP in the central complex, focusing on the ellipsoid body, a circular neuropil region, in control and Aβ42 fly brains at 21 dae. mito-GFP signals are shown as ratios relative to control (mean±SD, n = 3–5; *, p<0.05, Student's t-test). (D) Signal intensities of CD8-GFP in axon bundle tips and dendrites in control and Aβ42 fly lines at 21 dae are shown as ratios relative to control (mean±SD, n = 6–10). (E) Signal intensities of tubulin-GFP (tub-GFP) in axon bundle tips and dendrites in controls and Aβ42 fly lines at 26 dae are shown as ratios relative to control (mean±SD, n = 6–10). (F) Signal intensities of synaptotagmin-GFP (syt-GFP) in axon bundle tips and cell bodies in controls and Aβ42 fly lines at 24 dae are shown as ratios relative to control (mean±SD, n = 6–10). (C–E) No significant difference was detected between control and Aβ42 fly brains (p>0.05, Student's t-test). Male flies with the pan-neuronal elav-GAL4 driver were used for all experiments shown in Figure 1.

Using mito-GFP transgene, a reporter construct in which GFP is fused to a mitochondrial targeting signal [24], we analyzed the distribution of mitochondria in the Aβ42 fly brain. For this purpose, we focused on the mushroom body structure, where axons, dendrites, and cell bodies can be easily identified in the fly brain [25] (Figure 1A). Mito-GFP and Aβ42 were expressed in all neurons by the pan-neuronal elav-GAL4 driver.

The mito-GFP signal in the axons and dendrites of the mushroom body structure was significantly decreased in the Aβ42 fly brains (Figure 1B). In contrast, the mito-GFP signal was increased in the cell bodies of neurons (Figure 1B). These results suggest that Aβ42 does not cause global reduction of mitochondria, but rather induces mitochondrial mislocalization. A significant reduction in mitochondria was observed in the axons at 5 days after eclosion (dae), while a reduction in mitochondria in the dendrites was detected by 21 dae. Thus, Aβ42-induced reduction of mitochondria in the axons occurs earlier than in the dendrites. Similar results were obtained from four independent Aβ42 transgenic fly lines using the pan-neuronal elav-GAL4 driver (Figure 1B), or the cholinergic neuron-specific driver, Cha-GAL4 (Figure S1). Reduced mito-GFP signals in neuropil in Aβ42 fly brains were also observed in other brain structures including the central complex (Figure 1C), which is required for the maintenance of locomotor activity in flies [26].

Mitochondrial mislocalization observed in the Aβ42 fly brains is not due to overexpression of exogeneous protein, since neuronal expression human α-synuclein [27], which is thought to play a critical role in Parkinson's disease, did not induce mislocalization of mitochondria in the fly brains at 21 dae (Figure S2).

The reduction in mitochondria in the axons and dendrites is unlikely to be due to degeneration of the mushroom body structure, since neurodegeneration in the Aβ42 fly brain is not prominent at 5 dae [13], [14]. To confirm that the mushroom body structure has not degenerated, and to test whether Aβ42 expression non-specifically alters the protein distribution in axons and dendrites, we analyzed the distribution of the membrane protein CD8 fused to a GFP reporter (CD8-GFP). Aβ42 did not cause a noticeable morphological change of the mushroom body structures or significant reduction in the CD8-GFP signal in axons or dendrites at 21 dae (Figure 1D).

Mitochondria are transported along microtubules by the motor proteins. To test whether Aβ42-induced mitochondrial mislocalization is due to an overall disruption of microtubule-based transport in neurons, we analyzed distributions of tubulin fused to GFP (tub-GFP) and the presynaptic protein synaptotagmin fused to GFP (syt-GFP) in axons and dendrites. Aβ42 expression did not result in any significant difference in the distributions of tub-GFP in axons and dendrites (Figure 1E) or syt-GFP in axons and cell bodies (Figure 1F) at 21 dae. These data suggest that Aβ42-induced mislocalization of mitochondria is not due to disorganization of microtubule or global disruption of axonal transport in neurons.

### Mitochondria Are Not Severely Damaged in Young Aβ42 Fly Brains

**Figure 2:**
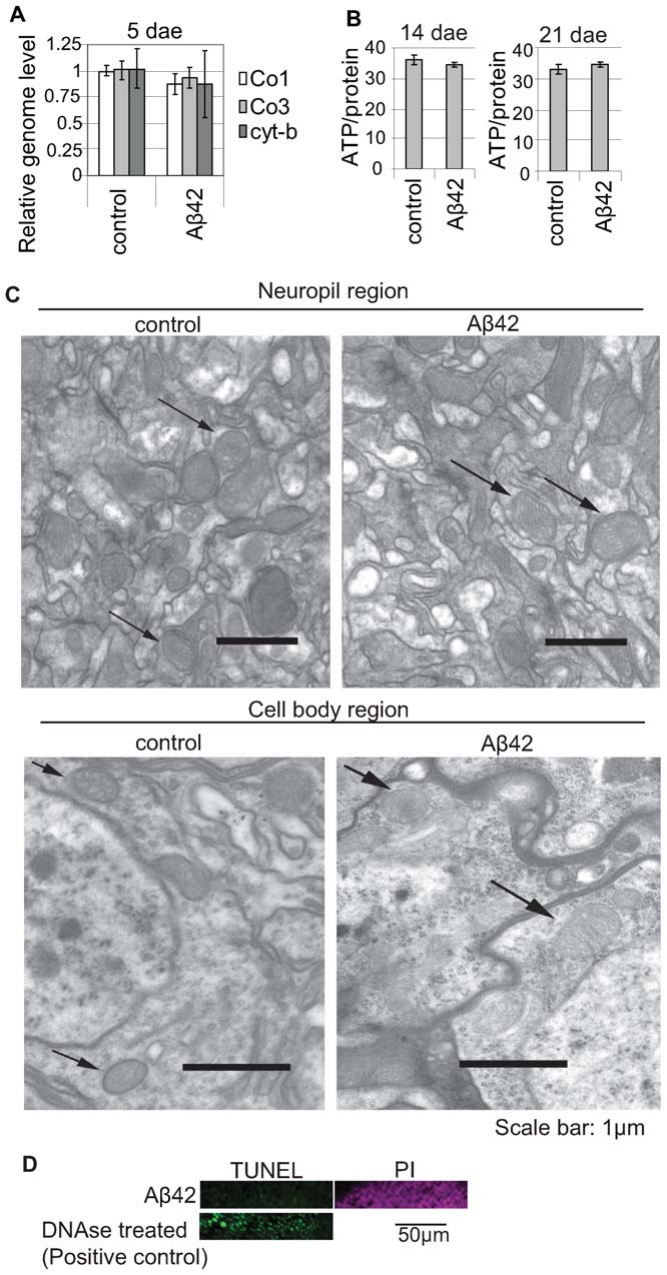
Mitochondria are not severely damaged in young Aβ42 fly brains. (A) Quantitation of mitochondrial DNA in the brains dissected from Aβ42 and control flies at 5 dae using quantitative Real Time-PCR. DNA levels are shown as ratios relative to control (mean±SD, n = 6). No significant difference was detected (p>0.05, Student's t-test). Co I, cytochrome c oxidase subunit I; Co III, cytochrome c oxidase subunit III, Cyt B, cytochrome *b*. (B) Quantitation of the ATP levels in the brains dissected from Aβ42 and control flies at 14 dae and 21 dae. ATP levels are normalized to the protein level (mean±SD). No significant difference was detected (n = 4; p>0.05, Student's t-test). (C) Transmission electron microscopic analysis of mitochondrial morphology in the neuropil (top) and cell body (bottom) regions of mushroom body structures in control and Aβ42 fly brains at 5 dae. (D) Aβ42 fly brains at 25 dae (Aβ42) did not contain TUNEL positive cells in Kenyon cell body region. Nuclei are labeled with Propidium Iodide (magenta). Brains treated with DNAse were used as a positive control (green). Male flies with the pan-neuronal elav-GAL4 driver were used for all experiments shown in Figure 2.

Mitochondrial damage and dysfunction have been shown to alter mitochondrial localization. We examined whether Aβ42 caused severe mitochondrial damage at the ages at which we observed mitochondrial mislocalization. We compared the amount of mitochondrial genomes and the levels of ATP in the brains dissected from control and Aβ42 flies, and found that they were not significantly different (Figure 2A and B). Electron microscopic (EM) analysis did not detect noticeable alterations in mitochondrial morphology in the neuropil or cell bodies of Kenyon cell region in the Aβ42 fly brain (Figure 2C). These data suggest that mitochondrial mislocalization is not due to severe damage to the mitochondria in the Aβ42 fly brain.

Apoptosis can cause mitochondrial fragmentation and fission/fusion defects, which can result in mitochondrial mislocalization [28]. Apoptosis was not detected in the Aβ42 fly brain by EM analysis [13] or TUNEL staining (Figure 2D), suggesting that the Aβ42-induced reduction in mitochondria in neurites is not due to cellular responses associated with apoptosis.

### Aβ42-Induced Locomotor Deficits Are Enhanced by Genetic Reduction of Mitochondrial Transport

**Figure 3:**
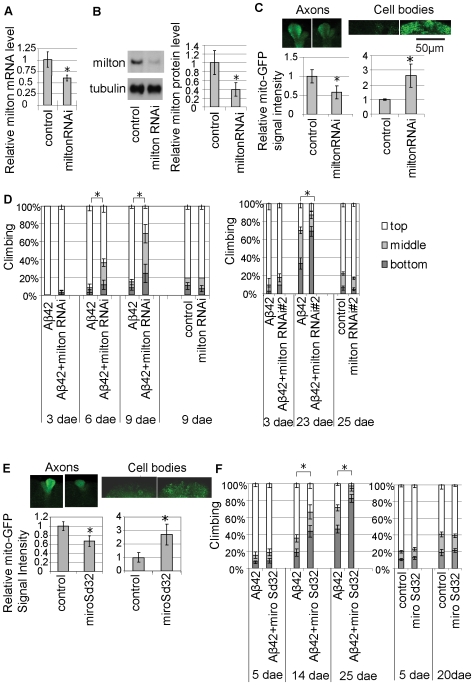
Aβ42-induced locomotor deficits are enhanced by genetic reductions of mitochondrial transport. (A) Neuronal expression of the RNAi transgene reduces milton mRNA levels. The milton mRNA levels in heads were quantified by qRT-PCR (n = 6; *p<0.01, Student's t-test). (B) Neuronal expression of the RNAi transgene reduces milton protein levels. The milton protein levels in brans were quantified by Western blotting with anti-*Drosophila* milton antibody. Signal intensities were quantified, normalized by tubulin levels, and are shown as ratios relative to control (mean±SD, n = 5; *p<0.05, Student's t-test). (C) Mislocalization of mitochondria in the mushroom body structure by neuronal knock-down of milton. Signal intensities of mito-GFP at 15 dae were quantified and are shown as ratios relative to control (mean±SD, n = 6; *, p<0.001, Student's t-test). (D) Enhancement of Aβ42-induced locomotor defects by neuronal knockdown of milton using UAS-RNAi transgenic fly lines. The average percentage of flies at the top (white), middle (light gray), or bottom (dark gray) of assay vials is shown (mean±SD, n = 5). Asterisks indicate the significant difference in the percentage of the flies stayed at the bottom (p<0.05, Student's t-test). (E) Mislocalization of mitochondria in the miro[Sd32] fly brain. Signal intensities of mito-GFP at 25 dae were quantified and are shown as ratios relative to control (mean±SD, n = 7–8; *, p<0.0001, Student's t-test). (F) Enhancement of Aβ42-induced locomotor defects in miro[Sd32] heterozygous background at 14 dae and 25 dae. Asterisks indicate the significant difference in the percentage of the flies stayed at the bottom (p<0.05, Student's t-test). Since both the elav-GAL4 and UAS-milton-RNAi are on X chromosome, female flies were used in Figure 3A, B, C, and the left panel in D. Male flies with the pan-neuronal elav-GAL4 driver were used in the right panel in D, E and F.

To test whether mitochondrial mislocalization contributes to Aβ42 toxicity, we examined the effect of a genetic reduction in mitochondrial transport on Aβ42-induced locomotor defects. Aβ42 flies show age-dependent, progressive locomotor dysfunction starting from 14 dae, which can be detected by climbing assay [13], [14]. In this assay, flies were placed in an empty plastic vial and tapped to the bottom. The number of flies at the top, middle, or bottom of the vial was scored after 10 seconds. Mitochondria are linked to motors by the mitochondrial membrane GTPase Miro, which is linked to kinesin by milton to allow transport in axons and dendrites [29]. Null mutations in *milton* and *Miro* have been reported to disrupt axonal and dendritic transport of mitochondria in neurons [30], [31]. Expression of milton RNAi in neurons with the pan-neuronal elav-GAL4 driver reduced the mRNA levels of milton in fly heads (Figure 3A), and resulted in 60% reduction in milton protein levels in dissected fly brains (Figure 3B). We analyzed mitochondrial localization in the mushroom body structures to confirm that milton RNAi expression caused a significant reduction in the mito-GFP signal in axons and an accumulation in somata (Figure 3C). Using this transgenic RNAi flies, we found that neuronal knockdown of milton enhanced Aβ42-induced locomotor defects, while milton knockdown itself did not cause locomotor defects at this age (Figure 3D, left). Similar results were obtained with the independent transgenic UAS-milton-RNAi fly line (Figure 3D, right).

A heterozygous *Miro* mutation (*miro[Sd32]*) also caused mitochondrial mislocalization (Figure 3E) and enhanced locomotor defects induced by Aβ42 (Figure 3F). Locomotor defects were not observed in the heterozygous *miro[Sd32]* mutant alone at 20 dae (Figure 3F). These results suggest that mitochondrial mislocalization contributes to Aβ42-induced behavioral deficits.

### Aβ42-Induced Locomotor Deficits Are Modified by cAMP Levels

**Figure 4:**
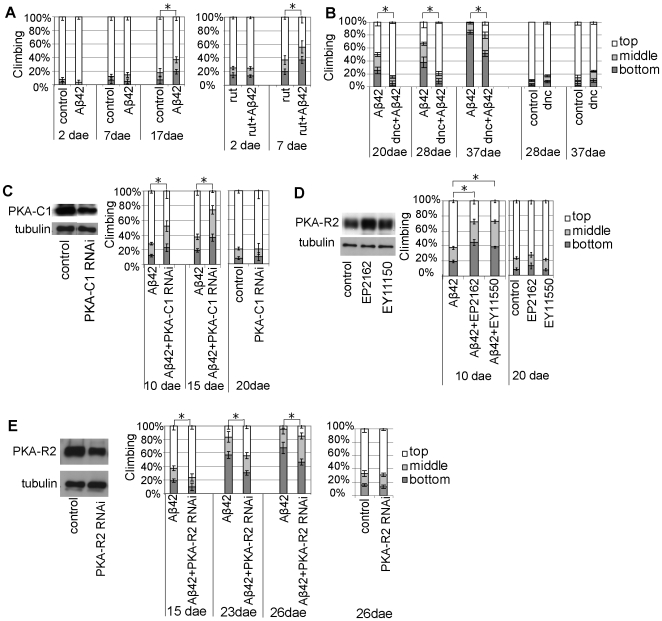
Aβ42-induced locomotor deficits are modified by neuronal cAMP levels and PKA activity. (A) Early onset of Aβ42-induced locomotor defects in the *rut* [*1*] background. The climbing ability of male flies expressing Aβ42 in the wild type (left) and in the *rut* [*1*] background (right). The Cha-GAL4 driver was used to drive Aβ42 expression. Male flies were used. (B) Aβ42-induced locomotor defects are suppressed in the *dnc* background. The Cha-GAL4 driver was used to drive Aβ42 expression. Male flies were used. (C) (Left) Neuronal expression of the RNAi transgene reduces PKA-C1 protein levels. Western blotting of PKA-C1 in fly head lysates. Tubulin was used to confirm equal protein loading in each lane. (Right) Enhancement of Aβ42-induced locomotor defects by neuronal knockdown of PKA-C1 (Aβ42+PKA-C1 RNAi). Neuronal knockdown of PKA-C1 by itself does not cause locomotor defects. Male flies with the pan-neuronal elav-GAL4 driver were used. (D) (Left) Increased PKA-R2 expression in EP2162 and EY11550 flies, as shown by Western blotting of PKA-R2 in fly head lysates. (Right) Enhancement of Aβ42-induced locomotor defects by overexpression of PKA-R2. PKA-R2 overexpression by itself does not cause locomotor defects. Male flies with the pan-neuronal elav-GAL4 driver were used. (E) (Left) Neuronal expression of the RNAi reduces PKA-R2 protein levels, as indicated by Western blotting of PKA-R2 in fly head lysates. (Right) Amelioration of Aβ42-induced locomotor defects by neuronal knockdown of PKA-R2 (PKA-R2 RNAi). PKA-R2 RNAi by itself does not alter climbing ability. Male flies with the pan-neuronal elav-GAL4 driver were used. Asterisks indicate the significant difference in the percentage of the flies stayed at the bottom (mean±SD, n = 5, p<0.05, Student's t-test).

cAMP is generated from ATP, and depletion of mitochondria in axons has been shown to disrupt cAMP/PKA signaling, which limits mobilization of the synaptic vesicle reserve pool in presynaptic terminals, and reduces synaptic strength [32]. We tested whether a reduction in the cAMP level by a genetic reduction of the *rutabaga*-encoded type I Ca^2+^/CaM-dependent adenylyl cyclase (rut) enhanced neuronal dysfunction in Aβ42 flies. Since the *rutabaga* mutation (*rut[1]*) is X-linked, we used the cholinergic neuron-specific Cha-GAL4 driver on the second chromosome, instead of the pan-neuronal elav-GAL4 driver on X choromosome, to drive Aβ42 expression in male flies in the *rut[1]* background. Expression of Aβ42 in cholinergic neurons using the Cha-gal4 driver caused locomotor defects by 17 dae (Figure 4A, left). In contrast, in the *rutabaga* mutant background (*rut[1]*), Aβ42 caused locomotor dysfunctions by 7 dae (Figure 4A, right). Thus, reduced cAMP levels result in an earlier onset of Aβ42-induced locomotor defects.

Next, we tested whether an increase in the cAMP level by a genetic reduction of the *dunce*-encoded phosphodiesterase (PDE), an enzyme that degrades cAMP, ameliorated neuronal dysfunction in Aβ42 flies. Since the *dnc* mutation (*dnc[1]*) is also X-linked, we used the cholinergic neuron-specific Cha-GAL4 driver on the second chromosome to drive Aβ42 expression in male flies in *dnc[1]* mutant background. We found that Aβ42-induced locomotor defects were suppressed in flies with a hypomorphic mutation of *dnc* (*dnc[1]*). In contrast, *dnc[1]* flies show similar locomotor function as the control flies (See the “material and methods” section for genetic background for *dnc[1]* and control flies) (Figure 4B).

### Aβ42-Induced Locomotor Defects Are Modified by Neuronal PKA Activity

Since PKA activity is regulated by cAMP levels, we examined whether PKA activity is involved in Aβ42-induced toxicity. Knockdown of the catalytic subunit of PKA (PKA-C1) in neurons using UAS-PKA-C1-RNAi driven by the pan-neuronal elav-GAL4 driver enhanced Aβ42-induced locomotor defects, while neuronal knockdown of PKA-C1 by itself did not cause locomotor defects at this stage (Figure 4C).

PKA activity is suppressed by binding of the regulatory subunits (PKA-R) to the catalytic subunit, and overexpression of PKA-R decreases, while knockdown of PKA-R increases, PKA activity. The transgenic fly lines EP2162 and EY11550 overexpress PKA-R2 in neurons when combined with the pan-neuronal elav-GAL4 driver. We found that neuronal overexpression of PKA-R2 significantly enhanced Aβ42-induced locomotor defects, while overexpression of PKA-R2 by itself did not affect locomotor function (Figure 4D).

We further examined the effects of a reduction in neuronal PKA-R2 expression on Aβ42-induced locomotor dysfunctions. Knockdown of PKA-R2 in neurons using an RNAi transgene with the pan-neuronal elav-GAL4 driver suppressed the locomotor defects in Aβ42 flies, while PKA-R2 knockdown by itself did not affect locomotor function (Figure 4E). Similar results were observed using an independent Aβ42 transgenic fly line (Figure S3).

Because rut, dnc, and the PKA complex is enriched in the axons and dendrites in fly neurons [33], these results suggest that neuronal dysfunctions in Aβ42 flies may be attributable to reduced cAMP/PKA signaling in the axons and dendrites.

Neuronal knock-down of PKA-C1 or PKA-R2 did not affect the accumulation of Aβ42 (Figure S4), the number of Aβ42 aggregation detected as Thioflavin S-positive deposits (Figure S5), or neurodegeneration (Figure S6) in the Aβ42 fly brain.

### The Levels of Putative PKA Substrate Phosphoproteins Are Reduced in the Aβ42 Fly Brain

**Figure 5:**
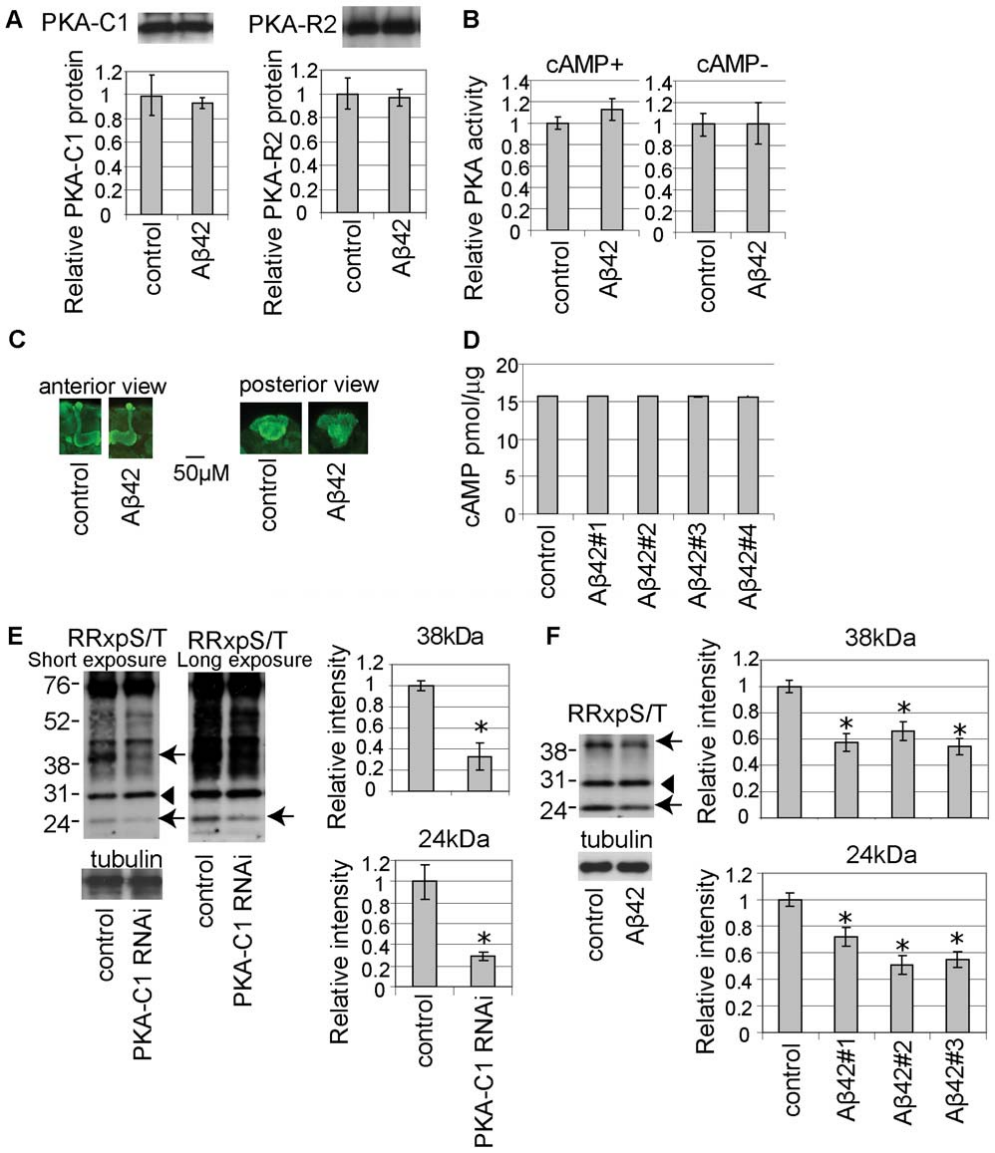
The levels of putative PKA substrate phosphoproteins are reduced in the Aβ42 fly brain. (A) Total protein levels of PKA-C1 (Left) or PKA-R2 (Right) in Aβ42 fly brains. Western blot using anti-PKA-C1 or anti-PKA-R2. Signal intensities were quantified and are shown as ratios relative to control (mean±SD, n = 5; p>0.05, Student's t-test). (B) PKA activity in brain extracts from control or Aβ42 flies at 21 dae. The PKA activity was normalized to the protein level and is shown as a ratio relative to control. No significant differences were observed (n = 4; p>0.05, Student's t-test). (C) Whole-mount immunostaining of brains of control and Aβ42 flies at 14dae using anti-PKA-C1 (green). (D) cAMP in head extracts from control flies and Aβ42 fly lines at 25 dae. The amount of cAMP was normalized to total protein. No significant differences were observed (n = 6; p>0.05, Student's t-test). A representative standard curve and control experiment are shown in Figure S7. (E) Identification of 24 kDa and 38 kDa proteins (arrows) as potential PKA substrates in the fly brain. Western blotting of brain lysates from control or PKA-C1 neuronal knockdown flies at 8 dae using a phospho-PKA substrate antibody (anti-RRxpS/T). The signal intensities were normalized to the tubulin level and are shown as ratios relative to controls. Asterisks indicate significant differences from control (n = 4; *, p<0.05, Student's t-test). The signal intensity of the 30 kDa protein (arrowhead) was not reduced (n = 4; p>0.05, Student's t-test). (F) The signal intensities of the 24 kDa and 38 kDa proteins (arrows), but not the 30 kDa protein (arrowhead), were reduced in brain extracts of Aβ42 fly lines (Aβ42-#1–3) at 8 dae. Asterisks indicate significant differences from control (n = 4; *, p<0.05, Student's t-test). Male flies with the pan-neuronal elav-GAL4 driver were used in all experiments in Figure 5.

To test whether PKA activity is reduced in the Aβ42 fly brain, we compared PKA-C1 and PKA-R2 protein levels and total PKA activity in extracts from dissected brains from Aβ42 and control flies. These parameters were not significantly different (Figure 5A and 5B). We next examined whether the cellular distribution of PKA is altered in the Aβ42 fly brain by immunostaining. A strong PKA-C1 signal was detected in the axons and dendrites, with less staining in the cell bodies of mushroom body structure. We did not detect obvious differences between Aβ42 and control fly brains (Figure 5C). We also compared that cAMP levels in head extracts of Aβ42 and control flies, and found they were not significantly different (Figure 5D and Figure S7).

To further investigate whether the levels of putative PKA substrate phosphoproteins are reduced in the Aβ42 fly brain, we performed Western blot analysis using a phospho-PKA substrate antibody (anti-RRxpS/T). We first identified the signals whose reductions were correlated with the decreased PKA activity in the dissected fly brains. Neuronal knockdown of PKA-C1 markedly reduced the signal intensities of phosphoproteins migrating at 24 kDa and 38 kDa (Figure 5E, arrows). Because the identity of the 24 kDa and 38 kDa proteins is currently under investigation, it is not clear whether phosphorylation of these proteins is decreased without changes in the steady-state levels, and these proteins may be phosphorylated by kinases other than PKA. Nevertheless, since neuronal knockdown of PKA-C1 markedly reduced these signals, the levels of 24 kDa and 38 kDa phosphoproteins are correlated with PKA activity in the fly brains. We also found that some of the signals detected by anti-RRxpS/T, including a protein migrating at 30 kDa, were not affected by PKA-C1 knockdown in fly brains (Figure 5E, arrowhead).

The signals of the 24 kDa and 38 kDa phosphoproteins were significantly reduced in the brains dissected from three independent Aβ42 fly lines (Figure 5F, arrows). In contrast, the signal of the 30 kDa protein was not affected by Aβ42 expression (Figure 5F, arrowhead). Although we did not detect a change in overall PKA activity or cAMP levels, these data suggest that cAMP/PKA signaling is disrupted in the Aβ42 fly brain.

**Figure 6:**
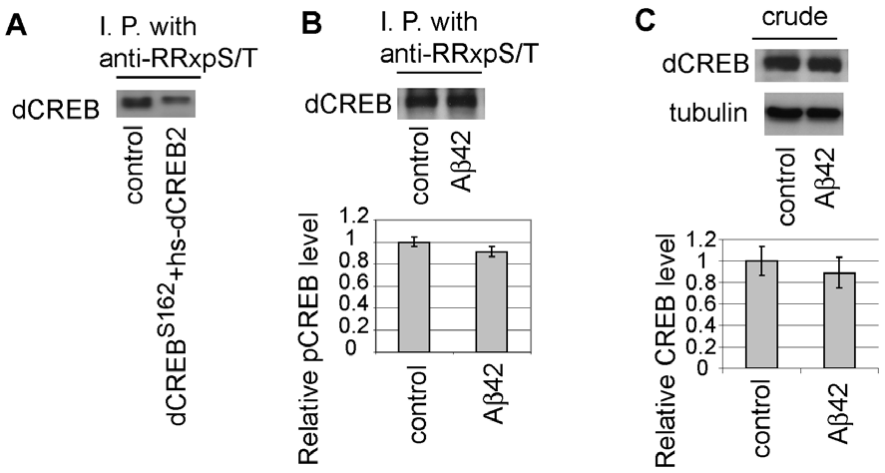
Phosphorylation of dCREB is not reduced in head extracts from Aβ42 flies at 25 dae. (A) Anti-RRxpS/T detects phosphorylation of *Drosophila* CREB (dCREB) at Ser231. dCREB Ser231, the site equivalent to Ser133 of mammalian CREB, is the only RRxpS/T site in dCREB. Head extracts were subjected to immunoprecipitation using anti-RRxpS/T, followed by Western blotting with anti-dCREB. The specificity of the antibody was confirmed using loss-of-function dCREB mutant flies (CREB^S162^). A low level of expression of a dCREB transgene was used to rescue lethality of CREB^S162^ (CREB^S162^+hs-dCREB) [59]. The signal detected by anti-dCREB was reduced in these flies. (B) Head extracts from control flies or from Aβ42 flies at 25 dae were subjected to immunoprecipitation using anti-RRxpS/T, followed by Western blotting with anti-dCREB. The phosphorylated CREB levels were normalized by the CREB level detected by Western blotting of the crude extract and are shown as ratios relative to controls. No difference in phosphorylated dCREB signal was detected (mean±SD, n = 4; p>0.05). (C) The total CREB level is not altered in Aβ42 fly brains. The CREB levels were normalized by the tubulin levels detected by Western blotting and are shown as ratios relative to controls. (mean±SD, n = 4; p>0.05).

In mammals, PKA activates cAMP-response element binding protein (CREB) via direct phosphorylation at Ser133, and *Drosophila* CREB (dCREB) Ser231 is equivalent to the mammalian Ser133 [34]. dCREB migrates around 38 kDa, and a reduction in CREB phosphorylation has been reported in cellular and animal models of AD [35], [36]. We tested whether anti-RRxpS/T recognized phopshorylated dCREB, and whether phosphorylation of dCREB was affected by Aβ42 expression by immunoprecipitation with anti-RRxpS/T followed by Western blotting with anti-dCREB antibody. Anti-RRxpS/T recognized phosphorylated dCREB (Figure 6A), while we did not detect a significant reduction in dCREB phosphorylation in the Aβ42 fly brain (Figure 6B–C). This result indicates that a reduction of the level of 38 kDa phosphoprotein in the Aβ42 fly brain is not due to a reduction in phosphorylation of dCREB.

### Disruption of Mitochondrial Transport Causes Age-Dependent Behavioral Deficits and Reduces the Levels of Putative PKA Substrate Phosphoproteins

**Figure 7:**
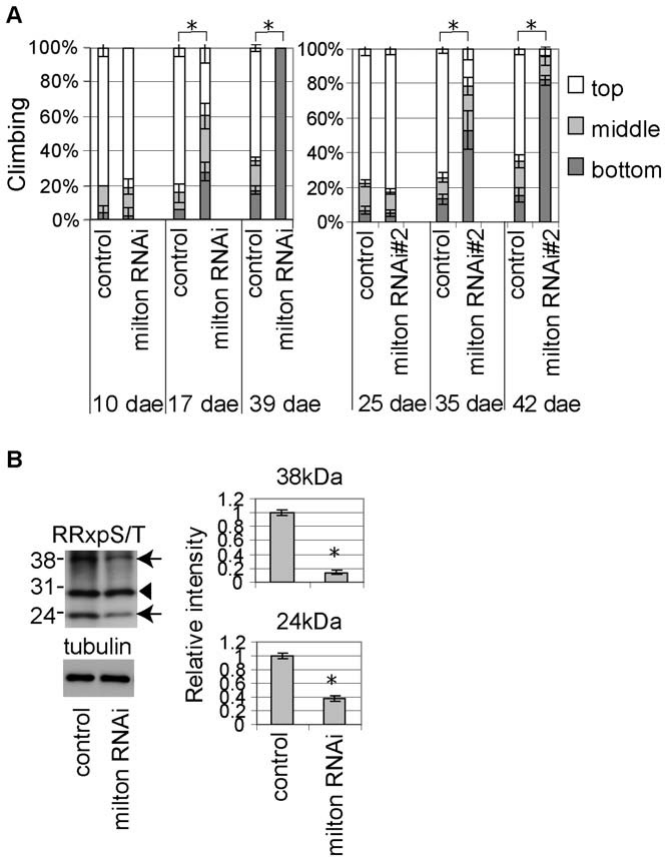
Disruption of mitochondrial transport causes age-dependent behavioral deficits and reduces the levels of putative PKA substrate phosphoproteins. (A) Age-dependent locomotor defects in the flies with a neuronal knockdown of milton using UAS-milton RNAi transgenic fly lines. Asterisks indicate the significant difference in the percentage of the flies stayed at the bottom (mean±SD, n = 5, p<0.05, Student's t-test). (B) The signal intensities of the 24 kDa and 38 kDa (arrows) proteins, but not the 30 kDa (arrowhead) protein, were reduced in brain extracts of flies with a neuronal knockdown of milton at 8 dae. The signal intensities were normalized to the tubulin level and are shown as ratios relative to controls. Asterisks indicate significant differences from control (n = 4; *, p<0.05, Student's t-test). The pan-neuronal elav-GAL4 driver was used. Since both the elav-GAL4 and UAS-milton-RNAi are on X chromosome, female flies were used in the left panel in A and B. Male flies with the elav-GAL4 driver were used in the right panel in A.

We have shown that mitochondria are reduced in the axons and dendrites in the Aβ42 fly brain (Figure 1) and that a genetic reduction in mitochondrial transport enhances Aβ42-induced behavioral deficits (Figure 3). We examined whether a disruption in mitochondrial transport is sufficient to cause late-onset behavioral deficits. Neuronal knockdown of milton by UAS-milton-RNAi driven by the pan-neuronal elav-GAL4 driver did not affect locomotor function up to 10 dae (Figure 7A, left), but caused locomotor dysfunctions after 17 dae (Figure 7A, left). Similar results were obtained with the independent UAS-milton-RNAi transgenic fly line (Figure 7A, right). In addition, we found that the levels of the 24 kDa and 38 kDa phosphoproteins were reduced in the brains dissected from flies with neuronal knockdown of milton (Figure 7B, arrows), suggesting that mitochondrial mislocalization causes disruption of cAMP/PKA signaling.

## Discussion

Elucidation of mechanisms underlying Aβ42-induced toxicities is crucial to understanding the complex pathogenesis of AD. An altered distribution of mitochondria has been reported in the brains of AD patients and in cellular and animal models of Aβ toxicity [5], [6], [7], [8]. Using a transgenic *Drosophila* model, we have demonstrated that mislocalization of mitochondria is induced by Aβ42 without severe mitochondrial damage or neurodegeneration, and that mitochondrial mislocalization underlies neuronal dysfunction induced by Aβ42. Our findings suggest that mitochondrial mislocalization may contribute to the pathogenesis of AD.

### Mechanisms Underlying Aβ42-Induced Mitochondrial Mislocalization

In Aβ42 fly brains, Aβ42 is accumulated intraneuronally and extracellulary [14]. Although it is not yet clear whether intracellular and/or extracellular Aβ42 causes mitochondrial mislocalization, several possible mechanisms could underlie mitochondrial mislocalization in Aβ42 fly brain neurons.

Some reports have shown that Aβ is present within mitochondria and induces mitochondrial damage [7]. Damaged mitochondria are normally transported from neurites to the cell body for repair or degradation in autophagosomes, and persistent mitochondrial damage induced by Aβ42 may cause a reduction in mitochondria in neurites as a result [37]. Indeed, in neurons in the AD brain, damaged mitochondria accumulate in autophagosomes in the neuronal cytoplasm [10], [11]. In addition, mitochondrial fragmentation occurs during apoptosis [28], which could be induced by Aβ.

In the Aβ42 fly brain, mitochondrial mislocalization occurred without severe mitochondrial damage (Figure 2). An immunoEM analysis did not detect Aβ42 accumulation in mitochondria in neurons in the Aβ42 fly brain [14]. Moreover, our EM analysis [13] and TUNEL staining (Figure 2) did not detect apoptosis in the Aβ42 fly brain. These results suggest that severe mitochondrial damage or apoptosis is not likely to be the primary mechanism underlying mitochondrial mislocalization in the Aβ42 fly brain.

In neurons, mitochondria undergo fission perinuclearly in the cell body and are transported along microtubule or actin bundles [24], and global disruption of microtubule-dependent transport may cause reduced mitochondria in the axons and dendrites. Axonal swellings that potentially block transport have been observed in AD mouse models and human AD brains [38], and disruption of axonal transport increases Aβ generation [38], [39], [40]. Ultrastructural studies reveal a loss of the normal microtubular architecture near intracellular Aβ oligomers, which would impair movement of vesicles and mitochondria [41]. In contrast, it has also been reported that Aβ rapidly impair mitochondrial transport without affecting mitochondrial function or the cytoskeleton in hippocampal neurons [42]. In the Aβ42 fly brain, mitochondrial mislocalizaton was observed without significant alterations in microtubule assembly (Figure 1). In addition, we did not detect significant changes in the distribution of synaptotagmin-GFP, a marker for synaptic vesicles (Figure 1). These results suggest that mitochondrial mislocalization in the Aβ42 fly brain is not due to an overall disruption of microtubule-based transport but may be due to an altered transport specific to mitochondria.

Mitochondrial transport is regulated by several intracellular signals. Elevation of intracellular Ca^2+^, which occurs in regions of high metabolic demand such as nerve terminals and postsynaptic specializations, arrests microtubule-based mitochondrial movement. Mitochondria are linked to motors by the mitochondrial membrane GTPase Miro [29], and a recent study shows that Miro mediates the Ca2^2+^-dependent arrest of mitochondria [43], [44], [45]. Since altered Ca^2+^ homeostasis is observed in AD neurons [46], [47], Aβ42 may impair mitochondrial movement by disruption of signaling that regulates mitochondrial transport to the axons and dendrites.

The motility of mitochondria is thought to be interrelated with the fission-fusion machinery and an imbalance between mitochondrial fission and fusion induced by Aβ42 may result in reduced mitochondria in the axons and dendrites [32], [48], [49]. In AD, mitochondrial size is increased and mitochondrial numbers are decreased in neurons [10], suggesting that the normally strict regulation of mitochondrial morphology is impaired. The dynamin-like GTPase, Drp1, influences mitochondrial density in axons and dendrites [32], [48], and reductions in Drp1 levels or increases in Drp1 activity by S-nitrosylation are induced by Aβ and detected in AD brains [49], [50], [51]. Very recent studies showed that mitochondria are reduced in neuronal processes in AD neurons [49], presumably due to alterations in the mitochondrial fission/fusion [49], [50], [51]. Although we did not detect severe changes in the morphology of mitochondria in the young Aβ42 fly brain (Figure 2), further morphometric analysis will be required to determine whether defects in fission and fusion occur and contribute to the mislocalization of mitochondria in the Aβ42 fly brain.

### Dysregulation of the cAMP/PKA Pathway in AD

Impaired regulation of the cAMP/PKA pathway has been reported in the brains of AD patients. Decreases in levels of specific adenylyl cyclase (AC) isoforms and disruption of AC/cAMP signal transduction have been detected in AD brains [52]. Decreased levels of the catalytic and regulatory subunits of PKA, as well as PKA activity, have also been observed in the brains of AD patients [53], although other reports did not detect widespread changes in PKA levels and activity in AD brains [52]. In cellular and animal models, Aβ causes an accumulation of PKA-R by reducing proteosomal degradation, which leads to a reduction in PKA activity [36]. In the Aβ42 fly brain, levels of putative PKA substrate phosphoproteins were significantly reduced, however, no overall changes in PKA protein levels, PKA activity, cAMP levels, or intracellular localization of the PKA complex were detected (Figure 5). cAMP is generated from ATP, and it has been shown that depletion of mitochondria in axons disrupt cAMP/PKA signaling [32].

Our results suggest that Aβ42-induced mitochondrial mislocalization causes local, but not global, alterations in cAMP/PKA activity, such as in the axons and dendrites.

In addition to cAMP/PKA signaling, the loss of ATP caused by the reduction of mitochondria in neurites could disrupt many biological processes and lead to neuronal dysfunction. Other major functions of mitochondria in neurons includes the regulation of Ca^2+^, which is important for synaptic plasticity, and cell survival [37]. Indeed, disrupting mitochondrial transport diminishes neuronal resistance to NMDA (N-methyl-D-aspartic acid)-induced excitotoxicity [45]. Thus, mitochondrial mislocalization may contribute to multiple aspects of Aβ42 toxicity in the brain.

### Concluding Remarks

Our study demonstrates that mislocalization of mitochondria underlies Aβ42-induced toxicity *in vivo*. Several reports show that the loss of mitochondria from axons and dendrites is associated with defective synaptic transmission [30], [31], [32], [48]. AD begins as a disorder in synaptic function [54], which is believed to be associated with increased levels of Aβ42 in the brain [55]. Studies in animal models show that these functional deficits predate the onset of irreversible neurodegenerative damages [3], and restoration of the activities of certain signaling pathways could suppress Aβ42-induced neuronal dysfunctions. For example, rolipram, the most widely used PDE4 inhibitor, ameliorates memory impairments in APP-PSEN1 double transgenic mice [56]. Thus, interventions that rescue mitochondrial function, mitochondrial localization, and associated defects may maintain synaptic plasticity and neurological function [57]. Further studies of the physiological and pathophysiological mechanisms that affect mitochondrial localization may lead to novel approaches for the prevention and treatment of AD.

## Materials and Methods

### Fly Stocks and Antibodies

Transgenic fly lines carrying the human Aβ42 was established in the background of the Canton-S *w^1118^* (*isoCJ1*) genotype as described in [14]. The elav-GAL4^c155^ line was outcrossed with the *isoCJ1* flies for 5 generations. The X-linked *dnc* [*1*] allele, which was crossed into a background containing the *iso1CJ* autosomes, was a kind gift from Dr. T. Tully (Cold Spring Harbor Laboratory). A control cross to *iso1CJ* also was used. Other fly stocks and antibodies were obtained from: Drs. W. M. Saxton (UAS-mito-GFP, University of California, Santa Cruz), K. E. Zinsmaier (*miro[Sd32]*, The University of Arizona), L. Luo (UAS-CD8::GFP;;OK107, Stanford University), M. B. Feany (UAS-α synuclein, Harvard Medical School), the Bloomington *Drosophila* Stock Center (Indiana University) (elav-GAL4^c155^, gmr-GAL4, Cha-GAL4, UAS-tub-GFP, UAS-syt-GFP, *rut* [*1*], *PKA-R2 [EP2162]* and *PKA-R2 [EY11150]*), the VDRC stock center (UAS-milton RNAi flies (v41507 (labeled as #2) and v41508 (labeled as #1)), UAS-PKA-C1 RNAi (v6993) and UAS-PKA-R2 RNAi (v39436)) [58], T. L. Schwarz (anti-*Drosophila* milton, Harvard Medical School), and D. Kalderon (anti-PKA-C1 and anti-PKA-R2, Columbia University). A control cross to *w^1118^* from Bloomington Stock Center or *w^1118^* from VDRC was used for these flies. Anti-RRxpS/T (Cell Signaling, Beverly, MA) and anti-tubulin (Sigma, St. Louis, MO) were purchased.

### GFP Analysis in Fly Brains

Fly brains were dissected in cold PBS, fixed in PBS containing 4% paraformaldehyde (Electron Microscopy Sciences), and then placed under vacuum in PBS containing 4% paraformaldehyde and 0.25% Triton X-100. The fluorescence intensity in the mushroom body regions was analyzed using a confocal microscope (Carl Zeiss LSM 510) and quantified using NIH image.

### Genomic DNA Extraction and Quantitative Real Time PCR Analysis

Fly brains were dissected in cold PBS and frozen on dry ice, and genomic DNA was extracted. 20 brains were homogenated in 100 mM Tris-HCl pH 7.5, 100 mM EDTA, 100 mM NaCl, and 0.5% SDS, and incubate at 65°C for 30 min. Samples were treated with 1.5 M potassium acetate and 4 M LiCl, and incubated for 65°C for 30 min, and centrifuged. Supernatant was treated was phenol/chloroform, added isoprophanol, and centrifuged. Precipitated gemonic DNA was rinsed with 70% ethanol and subjected to quantitative real time-PCR (Applied Biosystems). The average threshold cycle value (Ct) was calculated from five replicates per sample. Levels of Co I, Co III and CytB DNA were standardized relative to that of rp49. Relative expression values were determined by the deltaCt method according to quantitative PCR Analysis User Bulletin (Applied Biosystems). Primers were designed using NIH primer blast as follows: Co I, CTGGAATTGCTCATGGTGGA (forward) and CTCCCGCTGGGTCAAAAA (reverse); Co III, CCCGCTATTGAATTAGGAGCA (forward) and ATTCCGTGGAATCCTGTTGC (reverse); CytB, TGAGGTGGATTTGCTGTTGA (forward) and TGGTTGAATATGGGCAGGTG (reverse); rp49, GCTAAGCTGTCGCACAAATG (forward) and GTTCGATCCGTAACCGATGT (reverse).

### ATP, PKA, and cAMP Assays

ATP contents in dissected brains without eye pigments were analyzed using ATP Bioluminescence Assay Kit CLSII (Roche, Mannheim). PKA activity in dissected brains without eye pigments was measured with MESACUP Protein Kinase Assay Kit (MBL, Woburn, MA) in the presence or absence of 2 µM cAMP. cAMP levels was measured with cAMP-screen system (Applied Biosystems, Foster City, CA). ATP, PKA and cAMP levels were calculated by standard curves and normalized by protein levels.

### Transmission Electron Microscopy

Probosces were removed from decapitated heads, which were then immersion-fixed overnight in 4% glutaraldehyde and 2% paraformaldehyde in 0.1 M PBS. Samples were post-fixed 1 hr in ferrocyanide-reduced osmium tetroxide (1% osmium tetroxide and 1.5% potassium ferrocyanide in distilled water). Fixation was followed by dehydration in a graded ethanol series and infiltration with Epon-Araldite resin (2 hr in 50% resin in acetone and 24 hr in 100% resin) using constant rotation. After transferring the samples to flat-bottom BEEM capsules with fresh resin, the samples were polymerized overnight at 60°C. Cured blocks containing fly heads were examined with a dissection microscope and heads with a suitable orientation (posterior oriented flat to the block surface) were selected for thin sectioning. Semi thin sections stained with toluidine blue were examined by light microscopy to localize the mushroom body region. Thin sections (120 nm) of entire heads were collected on nickel grids (100 mesh, Veco-EMS). Thin sections were stained for 5 minutes in lead citrate stain. Sections were examined and micrographs collected using a Hitachi H700T TEM.

### TUNEL Staining

Fly brains were fixed in PBS containing 4% paraformaldehyde (Electron Microscopy Sciences, Hatfield, PA), treated with 25 µg/ml proteinase K for 30 min, and incubated with In Situ Cell Death Detection Kit, Fluorescein (Roche, Mannheim) for 1 hr at 37°C. The brains were analyzed using a confocal microscope (Carl Zeiss LSM 510).

### RNA Extraction and Quantitative Real Time PCR Analysis

For each sample, 30–40 flies were collected and frozen. Heads were mechanically isolated, and total RNA was extracted using TRIzol (Invitrogen) according to the manufacturer's protocol with an additional centrifugation step (11,000×g for 10 min) to remove cuticle membranes prior to the addition of chloroform. Total RNA was reverse-transcribed using Superscript II reverse transcriptase (Invitrogen), and the resulting cDNA was used as a template for PCR on a 7500 fast real time PCR system (Applied Biosystems). The average threshold cycle value (Ct) was calculated from five replicates per sample. Expression of milton was standardized relative to actin. Relative expression values were determined by the deltaCt method according to quantitative PCR Analysis User Bulletin (Applied Biosystems). Primers were designed using NIH primer blast as follows: milton, CAGGATCAGCTGAAGCAACA (forward) and ACACGCTACCTCCCATTGTC (reverse); and actin5C, TGCACCGCAAGTGCTTCTAA G (forward) and TGCTGCACTCCAAACTTCCA (reverse).

### Western Blotting

Dissected brains were homogenized in Tris-glycine sample buffer (Invitrogen) and centrifuged at 13,000 rpm for 10 min, and the supernatants were separated on 6% or 10% Tris-glycine gels (Invitrogen) and transferred to nitrocellulose membranes (Invitrogen). The membranes were blocked with 5% nonfat dry milk (Nestlé) and blotted with the primary antibody (anti-*Drosophila* milton (a gift from Dr. T. L. Schwarz), anti-PKA-C1 (a gift from Dr. D. Kalderon), anti-PKA-R2 (a gift from Dr. D. Kalderon), anti-RRxpS/T (Cell Signaling), or anti-tubulin (Sigma)), incubated with appropriate secondary antibody and developed using ECL plus Western Blotting Detection Reagents (GE Healthcare).

### Climbing Assay

Climbing assay was performed as previously described [14]. Approximately 25 flies were placed in an empty plastic vial. The vial was gently tapped to knock the flies to the bottom, and the number of flies at the top, middle, or bottom of the vial was scored after 10 seconds. Experiments were repeated more than three times, and a representative result was shown.

### Whole-Mount Immunostaining

Fly brains were dissected in cold PBS, fixed in PBS containing 4% paraformaldehyde (Electron Microscopy Sciences), and then placed under vacuum in PBS containing 4% paraformaldehyde and 0.25% Triton X-100. After permeabilization with PBS containing 2% Triton X-100, the brains were stained with rabbit polyclonal anti-PKA-C1 antibody (a gift from Dr. D. Kalderon) followed by detection with biotin-XX goat anti-mouse IgG and streptavidin-Texas Red conjugate (Molecular Probes). The brains were analyzed using a confocal microscope (Carl Zeiss LSM 510).

### Sequential Extraction and Western Blotting of Aβ42

For sequential extractions of Aβ42, fly heads were homogenized in RIPA buffer (50 mM Tris-HCl, pH 8.0, 0.5% sodium deoxycholate, 1% Triton X-100, 150 mM NaCl) containing 1% SDS. Lysates were centrifuged at 100,000×g for 1 h, and supernatants were collected (SDS-soluble fraction). SDS-insoluble pellets were further homogenized in 70% formic acid (Sigma) followed by centrifugation at 13,000 rpm for 20 min, and the supernatants were collected (formic acid fraction). Formic acid was evaporated by SpeedVac (Savant, SC100), and protein was resuspended in dimethyl sulfoxide (Sigma). Protein extracts were separated on 10–20% Tris-Tricine gels (Invitrogen) and transferred to nitrocellulose membranes. The membranes were boiled in phosphate-buffered saline (PBS) for 3 min, blocked with 5% nonfat dry milk, blotted with the 6E10 antibody (Signet), incubated with appropriate secondary antibody and developed using ECL plus Western Blotting Detection Reagents (GE Healthcare).

### Thioflavin S Staining

For thioflavin S (TS) staining, the dissected brains were permeabilized and incubated in 50% EtOH containing 0.1% TS (Sigma) overnight. After washing in 50% EtOH and PBS, the brains were analyzed using a confocal microscope. The numbers of TS-positive deposits were quantified from four hemispheres from three flies per genotype. The fluorescence intensity in Kenyon cell regions was analyzed using a confocal microscope (Carl Zeiss LSM 510) and quantified using NIH image.

### Quantification of Neurodegeneration

For the analysis of neurodegeneration in Kenyon cell region, heads were fixed in 4% paraformaldehyde, processed to embed in paraffin blocks, and sectioned at a thickness of 6 µm. Sections were placed on slides, stained with hematoxylin and eosin (Vector Laboratories), and examined by bright field microscopy. To quantify neurodegeneration, images of the sections were captured, and the areas of the vacuoles were measured using NIH Image.

### Detection of Phosphorylated dCREB by Immunoprecipitation

*dCREB[S162]* and the transgenic hs-dCREB2d line are from Dr. J. C.- P. Yin. Fly heads were homogenized in RIPA buffer (50 mM Tris·HCl, pH 8.0/0.5% sodium deoxycholate/1% Triton X-100/150 mM NaCl) containing 1% SDS, centrifuged, and supernatant was collected. Protein extracts were diluted to 1∶10 with RIPA buffer and immunoprecipitated with the anti-RRxpS/T antibody (Cell Signaling, Beverly, MA), separated on 10% Tris-Glycine gel (Invitrogen), and blotted with the anti-dCREB antibody (a gift from Dr. J. C.- P. Yin).

## Supporting Information

Figure S1 Mitochondria are mislocalized in cholinergic neurons in the Aβ42 fly brain. Mito-GFP in axon bundle tips, dendrites, and cell bodies of cholinergic neurons in the mushroom body in control and Aβ42 fly brains. The Cha-GAL4 driver was used to express transgene in cholinergic neurons. Signal intensities in control and Aβ42 flies at 35 dae were quantified and are shown as ratios relative to control (mean ± SD, n = 6–10; *, p<0.05, Student's t-test). Representative images are shown at the top. Male flies were used.
(0.07 MB DOC)

Figure S2 α-synuclein did not cause significant alteration of mitochondria localization in the fly brain. Mito-GFP in axon bundle tips, dendrites, and cell bodies in the mushroom body in control and α-synuclein fly brains. Transgene expression was driven by the pan-neuronal elav-GAL4 driver. Signal intensities in control and α-synuclein flies at 20 dae were quantified and are shown as ratios relative to control (mean ± SD, n = 6–10; *, p<0.05, Student's t-test). Representative images are shown at the top. Male flies were used.
(0.08 MB DOC)

Figure S3 Modification of Aβ42-induced locomotor defects by PKA activity was confirmed in an independent Aβ42 transgenic line. (A) Enhancement of Aβ42-induced locomotor defects by neuronal knockdown of PKA-C1 (Aβ42+PKA-C1 RNAi). (B) Enhancement of Aβ42-induced locomotor defects by overexpression of PKA-R2. (C) Suppression of Aβ42-induced locomotor defects by neuronal knockdown of PKA-R2 (PKA-R2 RNAi). Transgene expression was driven by the pan-neuronal elav-GAL4 driver. The average percentage of flies at the top (white), middle (light gray), or bottom (dark gray) of the assay vials is shown (mean ± SD, n = 5). Asterisks indicate the significant difference in the percentage of the flies stayed at the bottom (p<0.05, Student's t-test). Male flies were used.
(0.05 MB DOC)

Figure S4 Accumulation of Aβ42 was not affected by neuronal knockdown of PKA-C1 or PKA-R2 in fly brains. The effect of neuronal knockdown of PKA-C1 (A) or PKA-R2 (B) on Aβ42 accumulation in fly brains. Transgene expression was driven by the pan-neuronal elav-GAL4 driver. Aβ42 in brains from flies at 25 dae in the detergent soluble (RIPA/1%SDS) or insoluble (70%FA) fraction was detected by Western blotting. Aβ42 levels were normalized to tubulin levels and are shown as ratios relative to controls. Representative blots are shown on the left, and means ± SD are plotted on the right. No significant differences were detected (n = 5; p>0.05, Student's t-test). Male flies were used.
(0.08 MB DOC)

Figure S5 The number of Thioflavin S-positive Aβ42-deposits was not affected by neuronal knockdown of PKA-C1. The effect of neuronal knockdown of PKA-C1 on the formation of Aβ42-deposits. Thioflavin S (TS) staining of Kenyon cell body regions of the brain of flies expressing Aβ42 in the presence or absence of a PKA-C1 knockdown at 25 dae. Aβ42 expression was driven by the pan-neuronal elav-GAL4 driver. The numbers of TS-positive deposits in the Kenyon cell body regions are presented as the mean ± SD. No significant difference was detected (n = 4; p>0.05, Student's t-test). Male flies were used.
(0.06 MB DOC)

Figure S6 Aβ42-induced neurodegeneration is not affected by neuronal knockdown of PKA-C1 or PKA-R2. The effect of neuronal knockdown of PKA-C1 or PKA-R2 on Aβ42-induced neurodegeneration in fly brains. Transgene expression was driven by the pan-neuronal elav-GAL4 driver. Representative images of Kenyon cell bodies in flies expressing Aβ42 alone (Top), Aβ42 and PKA-C1 RNAi (Middle), or Aβ42 and PKA-R2 RNAi (Bottom) at 28 dae are shown on the left. Neurodegeneration, as reflected by the presence of vacuoles, is indicated by the arrows. Percentages of the area lost in the cell body regions are shown as means ± SD (n = 7–9 hemispheres). No significant differences from controls were detected (p>0.05, Student's t-test). Male flies were used.
(0.21 MB DOC)

Figure S7 An example of standard curves and control experiments for cAMP assay. The cAMP levels were measured using the cAMP-Screen assay kit (Applied Biosystems) according to the manufacturer's instruction. This assay is a competitive ELISA. Low levels of cAMP result in a high signal, while high levels result in a low signal. (Top) An example of standard curves. (Bottom) An example of readings with fly head lysates. Notice that the well containing fly head lysates without anti-cAMP antibody produced very low signal.
(0.04 MB DOC)
