# Supplementary material for: Mitochondrial Mislocalization Underlies Aβ42-Induced Neuronal Dysfunction in a Drosophila Model of Alzheimer's Disease
Source: PLoS One. 2009 Dec 15;4(12):e8310. doi: 10.1371/journal.pone.0008310 (PMC2790372; doi:10.1371/journal.pone.0008310)
Supplement: Figure S1 — Mitochondria are mislocalized in cholinergic neurons in the Aβ42 fly brain. Mito-GFP in axon bundle tips, dendrites, and cell bodies of cholinergic neurons in the mushroom body in control and Aβ42 fly brains. The Cha-GAL4 driver was used to express transgene in cholinergic neurons. Signal intensities in control and Aβ42 flies at 35 dae were quantified and are shown as ratios relative to control (mean ± SD, n = 6–10; *, p<0.05, Student's t-test). Representative images are shown at the top. Male flies were used. (0.07 MB DOC) [file pone.0008310.s001.doc]

**Figure S1. Mitochondria are mislocalized in cholinergic neurons in the Aβ42 fly brain.**

Mito-GFP in axon bundle tips, dendrites, and cell bodies of cholinergic neurons in the mushroom body in control and Aβ42 fly brains. The Cha-GAL4 driver was used to express transgene in cholinergic neurons. Signal intensities in control and Aβ42 flies at 35 dae were quantified and are shown as ratios relative to control (mean ± SD, n=6-10; *, p<0.05, Student’s t-test). Representative images are shown at the top. Male flies were used.
