# Supplementary material for: Mitochondrial Mislocalization Underlies Aβ42-Induced Neuronal Dysfunction in a Drosophila Model of Alzheimer's Disease
Source: PLoS One. 2009 Dec 15;4(12):e8310. doi: 10.1371/journal.pone.0008310 (PMC2790372; doi:10.1371/journal.pone.0008310)
Supplement: Figure S2 — α-synuclein did not cause significant alteration of mitochondria localization in the fly brain. Mito-GFP in axon bundle tips, dendrites, and cell bodies in the mushroom body in control and α-synuclein fly brains. Transgene expression was driven by the pan-neuronal elav-GAL4 driver. Signal intensities in control and α-synuclein flies at 20 dae were quantified and are shown as ratios relative to control (mean ± SD, n = 6–10; *, p<0.05, Student's t-test). Representative images are shown at the top. Male flies were used. (0.08 MB DOC) [file pone.0008310.s002.doc]

**Figure S2. α-synuclein did not cause significant alteration of mitochondria localization in the fly brain.**

Mito-GFP in axon bundle tips, dendrites, and cell bodies in the mushroom body in control and α-synuclein fly brains. Transgene expression was driven by the pan-neuronal elav-GAL4 driver. Signal intensities in control and α-synuclein flies at 20 dae were quantified and are shown as ratios relative to control (mean ± SD, n=6-10; *, p<0.05, Student’s t-test). Representative images are shown at the top. Male flies were used.
