# Supplementary material for: Mitochondrial Mislocalization Underlies Aβ42-Induced Neuronal Dysfunction in a Drosophila Model of Alzheimer's Disease
Source: PLoS One. 2009 Dec 15;4(12):e8310. doi: 10.1371/journal.pone.0008310 (PMC2790372; doi:10.1371/journal.pone.0008310)
Supplement: Figure S3 — Modification of Aβ42-induced locomotor defects by PKA activity was confirmed in an independent Aβ42 transgenic line. (A) Enhancement of Aβ42-induced locomotor defects by neuronal knockdown of PKA-C1 (Aβ42+PKA-C1 RNAi). (B) Enhancement of Aβ42-induced locomotor defects by overexpression of PKA-R2. (C) Suppression of Aβ42-induced locomotor defects by neuronal knockdown of PKA-R2 (PKA-R2 RNAi). Transgene expression was driven by the pan-neuronal elav-GAL4 driver. The average percentage of flies at the top (white), middle (light gray), or bottom (dark gray) of the assay vials is shown (mean ± SD, n = 5). Asterisks indicate the significant difference in the percentage of the flies stayed at the bottom (p<0.05, Student's t-test). Male flies were used. (0.05 MB DOC) [file pone.0008310.s003.doc]

**Figure S3. Modification of Aβ42-induced locomotor defects by PKA activity was confirmed in an independent Aβ42 transgenic line.**

(A) Enhancement of Aβ42-induced locomotor defects by neuronal knockdown of PKA-C1 (Aβ42+PKA-C1 RNAi). (B) Enhancement of Aβ42-induced locomotor defects by overexpression of PKA-R2. (C) Suppression of Aβ42-induced locomotor defects by neuronal knockdown of PKA-R2 (PKA-R2 RNAi). Transgene expression was driven by the pan-neuronal elav-GAL4 driver. The average percentage of flies at the top (white), middle (light gray), or bottom (dark gray) of the assay vials is shown (mean ± SD, n=5). Asterisks indicate the significant difference in the percentage of the flies stayed at the bottom (p<0.05, Student’s t-test). Male flies were used.
