# Supplementary material for: Mitochondrial Mislocalization Underlies Aβ42-Induced Neuronal Dysfunction in a Drosophila Model of Alzheimer's Disease
Source: PLoS One. 2009 Dec 15;4(12):e8310. doi: 10.1371/journal.pone.0008310 (PMC2790372; doi:10.1371/journal.pone.0008310)
Supplement: Figure S4 — Accumulation of Aβ42 was not affected by neuronal knockdown of PKA-C1 or PKA-R2 in fly brains. The effect of neuronal knockdown of PKA-C1 (A) or PKA-R2 (B) on Aβ42 accumulation in fly brains. Transgene expression was driven by the pan-neuronal elav-GAL4 driver. Aβ42 in brains from flies at 25 dae in the detergent soluble (RIPA/1%SDS) or insoluble (70%FA) fraction was detected by Western blotting. Aβ42 levels were normalized to tubulin levels and are shown as ratios relative to controls. Representative blots are shown on the left, and means ± SD are plotted on the right. No significant differences were detected (n = 5; p>0.05, Student's t-test). Male flies were used. (0.08 MB DOC) [file pone.0008310.s004.doc]

**Figure S4.** **Accumulation of Aβ42 was not affected by neuronal knockdown of PKA-C1 or PKA-R2 in fly brains.**

The effect of neuronal knockdown of PKA-C1 (A) or PKA-R2 (B) on Aβ42 accumulation in fly brains. Transgene expression was driven by the pan-neuronal elav-GAL4 driver. Aβ42 in brains from flies at 25 dae in the detergent soluble (RIPA/1%SDS) or insoluble (70%FA) fraction was detected by Western blotting. Aβ42 levels were normalized to tubulin levels and are shown as ratios relative to controls. Representative blots are shown on the left, and means ± SD are plotted on the right. No significant differences were detected (n=5; p>0.05, Student’s t-test). Male flies were used.
