# Supplementary material for: Mitochondrial Mislocalization Underlies Aβ42-Induced Neuronal Dysfunction in a Drosophila Model of Alzheimer's Disease
Source: PLoS One. 2009 Dec 15;4(12):e8310. doi: 10.1371/journal.pone.0008310 (PMC2790372; doi:10.1371/journal.pone.0008310)
Supplement: Figure S5 — The number of Thioflavin S-positive Aβ42-deposits was not affected by neuronal knockdown of PKA-C1. The effect of neuronal knockdown of PKA-C1 on the formation of Aβ42-deposits. Thioflavin S (TS) staining of Kenyon cell body regions of the brain of flies expressing Aβ42 in the presence or absence of a PKA-C1 knockdown at 25 dae. Aβ42 expression was driven by the pan-neuronal elav-GAL4 driver. The numbers of TS-positive deposits in the Kenyon cell body regions are presented as the mean ± SD. No significant difference was detected (n = 4; p>0.05, Student's t-test). Male flies were used. (0.06 MB DOC) [file pone.0008310.s005.doc]

**Figure S5. The number of Thioflavin S-positive Aβ42-deposits was not affected by neuronal knockdown of PKA-C1.**

The effect of neuronal knockdown of PKA-C1 on the formation of Aβ42-deposits. Thioflavin S (TS) staining of Kenyon cell body regions of the brain of flies expressing Aβ42 in the presence or absence of a PKA-C1 knockdown at 25 dae. Aβ42 expression was driven by the pan-neuronal elav-GAL4 driver. The numbers of TS-positive deposits in the Kenyon cell body regions are presented as the mean ± SD. No significant difference was detected (n=4; p>0.05, Student’s t-test). Male flies were used.
