# Supplementary material for: Mitochondrial Mislocalization Underlies Aβ42-Induced Neuronal Dysfunction in a Drosophila Model of Alzheimer's Disease
Source: PLoS One. 2009 Dec 15;4(12):e8310. doi: 10.1371/journal.pone.0008310 (PMC2790372; doi:10.1371/journal.pone.0008310)
Supplement: Figure S6 — Aβ42-induced neurodegeneration is not affected by neuronal knockdown of PKA-C1 or PKA-R2. The effect of neuronal knockdown of PKA-C1 or PKA-R2 on Aβ42-induced neurodegeneration in fly brains. Transgene expression was driven by the pan-neuronal elav-GAL4 driver. Representative images of Kenyon cell bodies in flies expressing Aβ42 alone (Top), Aβ42 and PKA-C1 RNAi (Middle), or Aβ42 and PKA-R2 RNAi (Bottom) at 28 dae are shown on the left. Neurodegeneration, as reflected by the presence of vacuoles, is indicated by the arrows. Percentages of the area lost in the cell body regions are shown as means ± SD (n = 7–9 hemispheres). No significant differences from controls were detected (p>0.05, Student's t-test). Male flies were used. (0.21 MB DOC) [file pone.0008310.s006.doc]

**Figure S6. Aβ42-induced neurodegeneration is not affected by neuronal knockdown of PKA-C1 or PKA-R2.**

The effect of neuronal knockdown of PKA-C1 or PKA-R2 on Aβ42-induced neurodegeneration in fly brains. Transgene expression was driven by the pan-neuronal elav-GAL4 driver. Representative images of Kenyon cell bodies in flies expressing Aβ42 alone (Top), Aβ42 and PKA-C1 RNAi (Middle), or Aβ42 and PKA-R2 RNAi (Bottom) at 28 dae are shown on the left. Neurodegeneration, as reflected by the presence of vacuoles, is indicated by the arrows. Percentages of the area lost in the cell body regions are shown as means ± SD (n=7-9 hemispheres). No significant differences from controls were detected (p>0.05, Student’s t-test). Male flies were used.
