# Supplementary material for: Mitochondrial Mislocalization Underlies Aβ42-Induced Neuronal Dysfunction in a Drosophila Model of Alzheimer's Disease
Source: PLoS One. 2009 Dec 15;4(12):e8310. doi: 10.1371/journal.pone.0008310 (PMC2790372; doi:10.1371/journal.pone.0008310)
Supplement: Figure S7 — An example of standard curves and control experiments for cAMP assay. The cAMP levels were measured using the cAMP-Screen assay kit (Applied Biosystems) according to the manufacturer's instruction. This assay is a competitive ELISA. Low levels of cAMP result in a high signal, while high levels result in a low signal. (Top) An example of standard curves. (Bottom) An example of readings with fly head lysates. Notice that the well containing fly head lysates without anti-cAMP antibody produced very low signal. (0.04 MB DOC) [file pone.0008310.s007.doc]

**Figure S7. An example of standard curves and control experiments for cAMP assay.**

The cAMP levels were measured using the cAMP-Screen assay kit (Applied Biosystems) according to the manufacture’s instruction. This assay is a competitive ELISA. Low levels of cAMP result in a high signal, while high levels result in a low signal. (Top) An example of standard curves. (Bottom) An example of readings with fly head lysates. Notice that the well containing fly head lysates without anti-cAMP antibody produced very low signal.
